# Cleaning and Disinfecting Oval-Shaped Root Canals: Ex Vivo Evaluation of Three Rotary Instrumentation Systems with Passive Ultrasonic Irrigation

**DOI:** 10.3390/medicina59050962

**Published:** 2023-05-16

**Authors:** Ying Li, Zhengyang Wang, Pingping Bao, Tingting Meng, Meng Liu, Huixu Li, Ya Shen, Dayong Liu, Zhi Jia, He Liu

**Affiliations:** 1 Department of Endodontics, Tianjin Stomatological Hospital, School of Medicine, NanKai University, Tianjin 300041, China; liying9211@126.com (Y.L.); pingpingbao0226@163.com (P.B.); minzhentanmeng@163.com (T.M.); lhxdentist@126.com (H.L.); 2 Tianjin Key Laboratory of Oral and Maxillofacial Function Reconstruction, Tianjin 300041, China; 3 Department of Endodontics, School of Stomatology, Tianjin Medical University, Tianjin 300070, China; zhengyangwang2021@163.com (Z.W.); lm905926573@163.com (M.L.); dyliuperio@tmu.edu.cn (D.L.); 4 Division of Endodontics, Department of Oral Biological and Medical Sciences, Faculty of Dentistry, The University of British Columbia, Vancouver, BC V6T 1Z3, Canada; yashen@dentistry.ubc.ca

**Keywords:** endodontic disinfection, endodontic irrigation, oval-shaped root canal, passive ultrasonic irrigation, scanning electron microscope

## Abstract

*Background and Objectives*: Successful root canal treatment depends on the thorough removal of biofilms through chemomechanical preparation. This study aimed to investigate and compare the cleaning and disinfecting efficiency of oval-shaped root canals using XP-endo Shaper (XPS), ProTaper Next (PTN), and HyFlex CM (HCM) in combination with passive ultrasonic irrigation (PUI). *Materials and Methods*: Ninety extracted teeth were contaminated and randomly divided into three groups: XPS, PTN, and HCM. Each group was assigned to three subgroups: subgroup A (sterile saline), subgroup B (3% sodium hypochlorite and 17% ethylenediaminetetraacetic acid), and subgroup C (3% sodium hypochlorite, 17% ethylenediaminetetraacetic acid, and PUI). Bacterial sampling was conducted both from baseline samples and samples after chemomechanical preparation. Scanning electron microscopy (SEM) was used to evaluate the residue bacterial biofilms, hard tissue debris, and smear layers on the buccolingual walls of oval-shaped root canals. *Results*: When combined with sterile saline, XPS demonstrated a higher reduction of bacterial counts and was more effective in eradicating *Enterococcus faecalis* in the middle third of the canals compared to the other instruments (*p* < 0.05). Additionally, when used with antimicrobial irrigants, XPS was more effective in disinfecting the coronal third of the canals than the other instruments (*p* < 0.05). Furthermore, XPS reduced hard tissue debris more effectively in the middle third of canals than in the apical third (*p* < 0.05). *Conclusions*: XPS outperforms PTN and HCM in disinfecting oval-shaped root canals. Despite the fact that combining XPS and PUI improves cleaning and disinfecting, removing hard tissue debris from the critical apical area remains challenging.

## 1. Introduction

Bacterial biofilm infections that cause apical periodontitis are of great concern to dental practitioners [1,2]. The eradication of biofilms through chemomechanical preparation is crucial for successful root canal treatment. However, the intricate morphologies of root canals, such as irregular cross-sections, isthmus, and fins, pose a great challenge [1,2,3]. Oval-shaped cross-sections are frequently observed in root canals, with a high incidence reported, which are more common than circular cross-sections [4,5,6]. The traditional nickel titanium (NiTi) rotary instruments have a cutting geometry that is not optimal for these shapes. As a result, mechanical instrumentation of oval-shaped root canals using traditional rotary systems can result in the removal of too much dentine or leave certain areas untouched [1,7]. Over the past few years, endodontic files with a variety of designs and concepts have been developed to adapt to the complex morphology of root canals. However, the optimal chemomechanical preparation of oval-shaped canals is still a challenge to current rotary files used today [8].

The XP-endo Shaper (XPS; FKG Dentaire, La Chaux-de-Fonds, Switzerland) is a newly introduced single-file rotary instrument that can adapt to the original anatomy of root canals in three dimensions during instrumentation [8,9,10]. The XPS file has an apical diameter of 0.30 mm and an initial taper of 1%, which increases to 4% at body temperature due to the MaxWire alloy. At 20 °C, the XPS remains in its martensitic phase, maintaining a straight form. However, when inserted into the canal at body temperature, it transforms into a snake-like shape in its austenitic phase. This unique design allows the file to easily adapt to irregular areas by expanding and contracting in the canal [11,12]. ProTaper Next (PTN; Dentsply Sirona, Ballaigues, Switzerland) is a multi-file rotary system with variable tapers made from M-Wire technology. It has a rectangular cross-sectional design and features an off-centered rotation [13]. HyFlex CM (HCM; Coltene-Whaledent, Allstetten, Switzerland) is a multi-file rotary system produced using a special thermomechanical treatment that enhances its flexibility and cyclic fatigue resistance. This allows it to follow the anatomy of canals, especially curved canals [14]. XPS, PTN, and HCM have unique designs, alloys, and movement patterns that distinguish them from one another. PTN employs the conventional M-Wire alloy and displays a blend of austenite and R-phase, with the onset of R-phase occurring around 45 °C [15]. Its cross-sectional configuration and asymmetric movement enable it to effectively cut more canal walls. In contrast, XPS and HCM have undergone thermomechanical processes and share a triangular cross-section design and flexibility. However, XPS stands out with its envelope of motion, which enables it to adapt more effectively to the morphology of root canals. Overall, these three systems possess distinct characteristics for preparing root canals [16,17,18,19,20]. However, to date, there is no study that compares the effectiveness of these systems in cleaning and disinfecting oval-shaped root canals. Therefore, the primary objective of this study was to assess and compare the cleaning and disinfecting performance of XPS, PTN, and HCM in oval-shaped root canals.

During mechanical instrumentation of the root canal system, a smear layer or hard tissue debris can form on the root canal walls, potentially harboring bacterial pathogens and hindering disinfection, ultimately compromising the quality of the root canal sealing and obturation [20]. To improve the effectiveness of rotary instruments, endodontic irrigants such as sodium hypochlorite (NaOCl) and ethylenediaminetetraacetic acid (EDTA) are frequently used in root canal treatment, with passive ultrasonic irrigation (PUI) techniques employed to activate the irrigants. These methods have been shown to enhance cleaning and disinfection in previous studies [21,22,23,24]. At present, there is no available literature that investigates the effectiveness of XPS, PTN, and HCM when used in conjunction with PUI for cleaning and disinfecting oval-shaped root canals. Therefore, the secondary objective of this study was to evaluate the efficacy of cleaning and disinfecting bacterial biofilm, hard tissue debris, and the smear layer in oval-shaped root canals using XPS, PTN, and HCM with or without PUI.

## 2. Materials and Methods

### 2.1. Samples Size Calculation and Selection

The Medical Ethics Committees of Stomatological Hospital, Tianjin Medical University approved the protocol of this study (certificate number: TMUh MEC2019005). Maxillary and mandibular premolar teeth with single roots, extracted for reasons unrelated to this study, were chosen and stored in 4 °C sterile saline for assessment. Cone-beam computed tomography (CBCT) scans were performed using a Smart3D-X scanner (Carestream Dental, Atlanta, GA, USA) to assess root canal morphology and dimensions. The exposure settings for CBCT included a fixed potential difference of 100 kV and an adjusted tube current of 8 mA. The spatial resolution was 2.2 lp/mm, and the voxel size was 119 μm^3^. The field of view (FOV) was 16 × 10 cm^2^, and the exposure time was 12.5 s. CBCT visualization was carried out through SmartVPro version 1.0 software (Carestream Dental).

**Figure 1:**
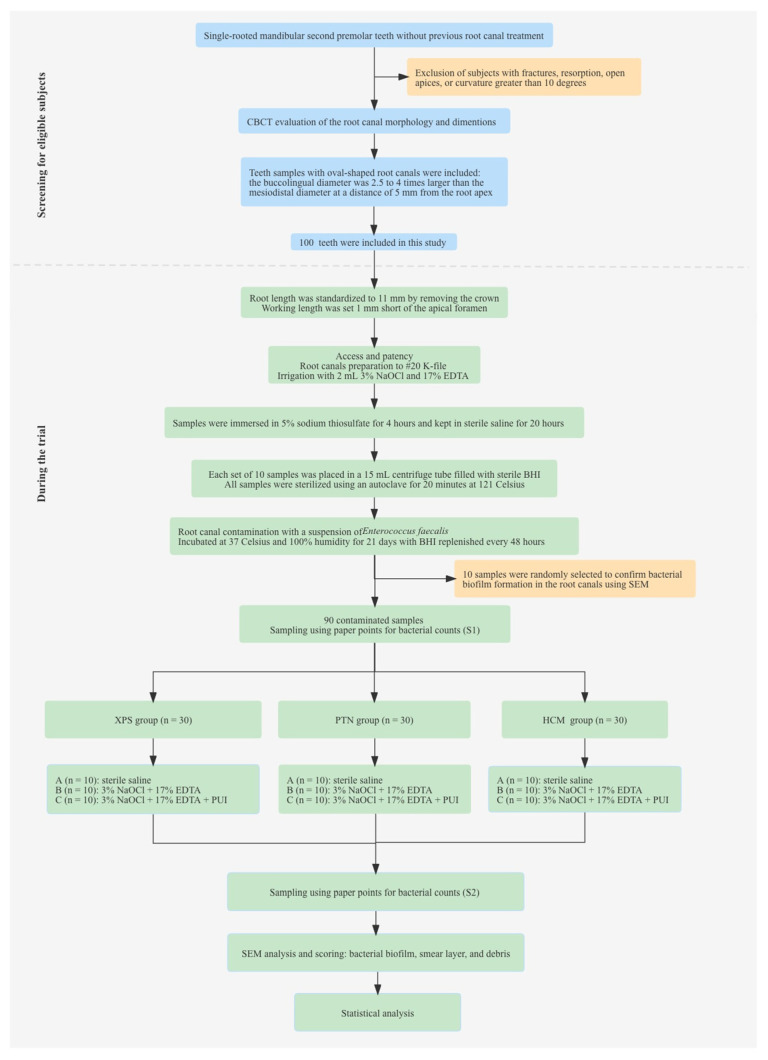
Flow diagram showing the selection of eligible subjects and the conduction of the trial. Abbreviations: BHI: brain heart infusion broth; CBCT: Cone-beam computed tomography; HCM: HyFlex CM; PTN: ProTaper Next; PUI: passive ultrasonic irrigation; SEM: scanning electron microscope; XPS: XP-endo Shaper.

The definition of oval-shaped root canals was based on previous studies [4,5]. Teeth with previous endodontic treatment, fractures, resorption, open apices, or a curvature greater than 10° were excluded. Prior to the experiment, a preliminary estimation was conducted using the PASS version 15 software (NCSS, Kaysville, UT, USA), which suggested that each subgroup should have a sample size of 7–9 teeth, with an inspection power of 95% and an alpha of 0.05. Finally, 100 teeth with a single, oval-shaped canal were included in this study. Figure 1 shows the selection of eligible teeth samples and the conduction of the trial.

### 2.2. Samples Preparation and Sterilization

The tooth length was standardized to 11 mm by removing the crown, using a high-speed diamond bur (Dentsply Maillefer, Ballaigues, Switzerland) with continuous water spray. The working length (WL) was established 1 mm short of the apical foramen using a size 10 K-file (Dentsply Maillefer), and the root canals were manually prepared with K-files up to size 20. During initial preparation, the canals were irrigated with 3% NaOCl (Vista Dental Products, Racine, WI, USA) using ProRinse^®^ 30-gauge side-vented needles (Dentsply Tulsa Dental Specialties, Tulsa, OK, USA). After preparation, the canals were irrigated with 17% EDTA (Biodynamics, Ibiporã, Brazil) for 1 min to remove the smear layer. The samples were then immersed in 5% sodium thiosulfate (Na_2_S_2_O_3_) (Sigma-Aldrich, St. Louis, MO, USA) for 4 h to inactivate any residual NaOCl on the root surface and within the canals. Subsequently, the teeth were kept in sterile saline for 20 h. After drying the canals with paper points (Dentsply Maillefer), the apical foramina were sealed with composite resin (3M Health Care, Saint Paul, MN, USA) to prevent apical bacterial leakage.

For each set of 10 specimens, a 15 mL centrifuge tube was filled with sterile brain heart infusion broth (BHI) (Merck KGaA, Darmstadt, Germany) and the samples were placed inside. The centrifuge tube was then agitated using a centrifuge (Intra-Lock, Boca Raton, FL, USA) for 30 s. All samples were sterilized using an autoclave (Hirayama, Kyoto, Japan) for 20 min at 121 °C. Following sterilization, the teeth were incubated at 37 °C for 48 h to verify the absence of any bacterial contamination.

### 2.3. Root Canal Contamination with Enterococcus faecalis

All samples were contaminated with *Enterococcus faecalis*. A 0.5 mL suspension of *E. faecalis*, at a concentration of 3 × 10^8^ colony-forming units (CFUs) per mL, was injected into a root canal that was placed in a 2 mL centrifuge tube containing 1 mL sterile BHI. The centrifuge tube was then fully agitated using a centrifuge for 30 s, resulting in an initial bacterial concentration of 1 × 10^8^ CFUs/mL in the canals. The specimens were then incubated at 37 °C and 100% humidity for 21 days, with the BHI replenished every 48 h. Ten specimens were randomly selected to confirm bacterial biofilm formation in the root canals using a FEI XL30 scanning electron microscope (SEM) (FEI Inc., Hillsboro, OR, USA). The SEM was operated with accelerating voltages of 5 kV, a working distance of 10 mm, and a 50 μm aperture. The resulting SEM images were visualized using SmartSEM software (Carl Zeiss AG, Oberkochen, Germany).

### 2.4. Root Canal Instrumentation and Bacterial Sampling

Bacterial sampling was conducted both from baseline samples (S1) and samples after chemomechanical preparation (S2). After incubation for 21 days, each canal was filled with 0.2 mL sterile saline solution, and bacterial sampling from S1 was sequentially conducted under strict asepsis using three sterile paper points at the WL for 1 min. The paper points were then transferred to an Eppendorf tube containing 1 mL sterile saline solution and agitated in a vortex (Vortex Genie 2, Scientific Industries, Bohemia, NY, USA) for 1 min. The bacterial suspension was subjected to serial dilutions, and different dilutions were then plated in triplicate on agar culture medium. The plates were then incubated at 37 °C for 48 h, after which the bacterial counts were determined in CFUs/mL.

This study involved ninety contaminated specimens that were separated into three groups in a randomized manner: XPS, PTN, and HCM. Each group was further separated into three subgroups (*n* = 10), each with distinct irrigation protocols: subgroup A (sterile saline), subgroup B (3% NaOCl and 17% EDTA), and subgroup C (3% NaOCl, 17% EDTA, and PUI). In the XPS group, XPS was inserted into the canals and activated with gentle lengthwise parietal movements for 3–5 strokes. After reaching the WL, XPS was used for an additional 10 gentle strokes along the entire length of the canal. In the PTN group, PTN was used with a crown-down technique, with the files used in sequence (X1, X2, and X3). In the HCM group, HCM was used in continuous rotation with the instruments used in sequence (20/0.04, 25/0.04, and 30/0.04). In subgroup A, the canal was irrigated with 2 mL sterile saline after each file. In subgroup B, the canal was rinsed with 2 mL of 3% NaOCl after each instrumentation and 5 mL of 17% EDTA after mechanical preparation was completed. In subgroup C, the irrigation procedure was similar to subgroup B, but supplemented with passive ultrasonic irrigation for 1 min at 2 mm short of the WL after the irrigation needle was removed from the canal. All of the irrigation was performed with 30-gauge side-vented irrigation needles. After chemomechanical preparation, the canals were irrigated with 5% Na_2_S_2_O_3_ to deactivate any remaining NaOCl. Bacterial sampling from S2 was conducted to evaluate the reduction in bacterial load.

### 2.5. Scanning Electron Microscope Analysis

The samples were fixed in a solution of 2.5% glutaraldehyde at 4 °C for 48 h. Subsequently, the samples were carefully longitudinally bisected into two halves to fully expose the buccolingual walls of the canals. The samples were then cautiously rinsed with phosphate-buffered saline and dehydrated with ascending concentrations of ethanol. Finally, the samples were mounted onto SEM disks, and a layer of gold was applied under vacuum coating. Using SEM at magnifications of 200×, 1000×, and 5000×, the residue hard tissue debris, smear layer, and bacterial biofilms on upper, middle, and bottom portions of the buccolingual walls of each canal were evaluated. The scores for bacterial biofilms, hard tissue debris, and the smear layer were recorded based on the criteria used in a previous study [25,26]. To evaluate these aspects, we assessed the upper, middle, and apical positions of the buccolingual walls of canals using three randomly selected fields of view per section. Each field was evaluated based on specific criteria, and the average score of the three fields was calculated to obtain the final score for each position of the sample.

For the biofilm score:

1. No bacteria on the surface of the root canal;
2. Isolated bacteria over the surface with no signs of viability/organization (mitosis, biofilm matrix);
3. Agglomeration of bacteria with signs of viability/organization (mitosis, biofilm, matrix);
4. More than 50% of the root canal walls were covered with viable bacteria;
5. Complete or nearly complete root canal wall coverage with viable bacteria.

(Bacterial biofilm was scored under a 5000× magnification.)

For the debris score:

1. Clean root canal wall, only a few small debris particles;
2. Few small agglomerations of debris, less than 25%;
3. Many agglomerations of debris covering less than 50% of the root canal wall;
4. More than 50% of the root canal wall covered by debris;
5. Complete or nearly complete root canal wall covered by debris, more then 75%.

(Scoring of debris was performed using a 200× magnification.)

For the smear layer score:

1. No smear layer, more then 90% dentinal tubules open;
2. Small amount of smear layer, some dentinal tubules open, more then 50%;
3. Homogenous smear layer covering the root canal wall, only few dentinal tubules open;
4. Complete root canal wall covered by a homogenous smear layer, less then 25% dentinal tubules open;
5. Heavy, nonhomogenous smear layer covering the complete root canal wall, no open dentinal tubule.

(Smear layer was scored under a 1000× magnification.)

### 2.6. Statistical Analysis

The data analysis was performed using SPSS 26.0 software (IBM SPSS Inc., Chicago, IL, USA). The bacterial counts were expressed as log10-transformation of the CFUs counts. To identify any differences among subgroups before instrumentation, we conducted a one-way analysis of variance (ANOVA) analysis on the S1 and S2 sampling for each group. After instrumentation, we observed a synergistic effect of the file system and irrigant method on the reduction in bacteria load. We analyzed the log-reduction data from S1 to S2 using a two-way ANOVA, after validating the normality and equal variance assumptions of the data sets. 

Three images acquired in same region were blindly evaluated by two calibrated examiners and the score was averaged. The differences in scores among the groups were analyzed using the Kruskal–Wallis test followed by a Tamhane T2 test at *p* < 0.05. A pre-defined statistical significance level of α = 0.05 was used for all analyses.

## 3. Results

**Figure 2:**
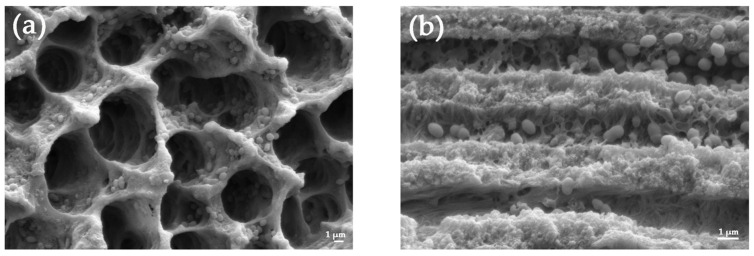
Representative scanning electron microscopy (SEM) images of *E. faecalis* colonization of the root canal before chemomechanical instrumentation. (**a**) A transverse section of dentinal tubules (3000×); (**b**) A longitudinal section of dentinal tubules (7000×). Bar = 1 μm.

*E. faecalis* cells had thoroughly inhabited the root canal walls, penetrating the dentinal tubules, and intermixing with viscous amorphous substances (Figure 2). The bacterial counts in S1 provided further evidence of successful root canal contamination.

### 3.1. Bacterial Counts before and after Chemomechanical Preparation

No significant differences were observed in the bacterial counts of S1 between the XPS, PTN, and HCM groups. After chemomechanical preparation, the bacterial counts significantly decreased in all groups from S1 to S2 (*p* < 0.05) (Table 1). The use of sterile saline resulted in a significantly lower percentage of bacterial counts reduction in PTN and HCM groups in comparison to the XPS group (*p* < 0.05) (Table 2). However, the use of antimicrobial irrigants (3% NaOCl and 17% EDTA) or PUI did not result in significant differences in the reduction of bacterial counts among the three groups. 

### 3.2. Assessment of the Effectiveness in Eliminating Bacterial Biofilm

**Figure 3:**
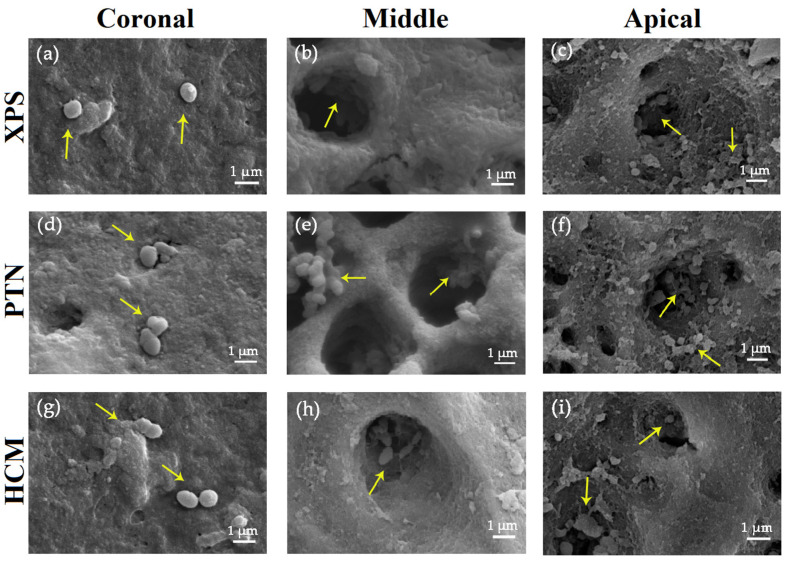
Representative SEM images of the buccolingual canal walls after rotary instrumentation in the coronal, middle, and apical thirds. The images were taken at a magnification of 5000×, and yellow arrows indicate bacteria residue. In the whole group, only isolated bacteria were observed on the canal wall surface in the coronal thirds (**a**,**d**,**g**). In all groups, bacterial residue on the buccolingual canal walls in the coronal and middle thirds were lower than the apical third. In the middle third, a few agglomerating bacteria with signs of viability were observed in the dentinal tubules of all three groups (**b**,**e**,**h**), whereas in the apical third, the bacterial density was higher, with more agglomerating bacteria in the tubules (**c**,**f**,**i**). Bars = 1 μm. Abbreviations: HCM: HyFlex CM; PTN: ProTaper Next; SEM: scanning electron microscope; XPS: XP-endo Shaper.

**Figure 4:**
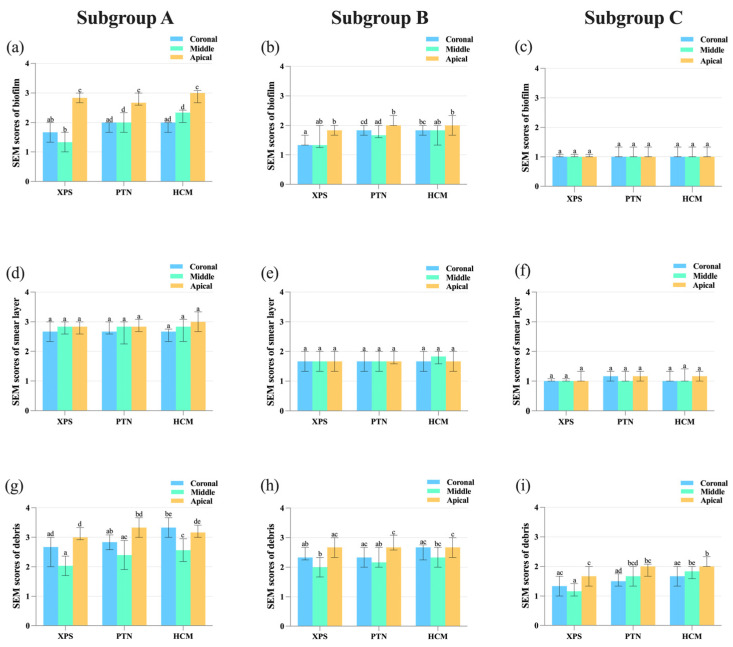
The bar charts showing SEM scores of bacterial biofilms (**a**–**c**), smear layer (**d**–**f**), and hard tissue debris (**g**–**i**) on the buccolingual walls of the root canal in the coronal, middle, and apical thirds, after chemomechanical preparation with different irrigants and rotary systems. (**a**,**d**,**g**) Subgroup A: Sterile saline; (**b**,**e**,**h**) Subgroup B: 3% NaOCl and 17% EDTA; (**c**,**f**,**i**) Subgroup C: 3% NaOCl, 17% EDTA, and PUI. Different lowercase letters indicate a significant difference at *p* < 0.05. Abbreviations: HCM: HyFlex CM; PTN: ProTaper Next; PUI: passive ultrasonic irrigation; SEM: scanning electron microscope; XPS: XP-endo Shaper.

During mechanical instrumentation, when the canals were irrigated with sterile saline, SEM images indicated a significant decrease in biofilm scores of the buccolingual canal wall in the middle section for the XPS group compared to the other groups (*p* < 0.05) (Figure 3 and Figure 4a). 

**Figure 5:**
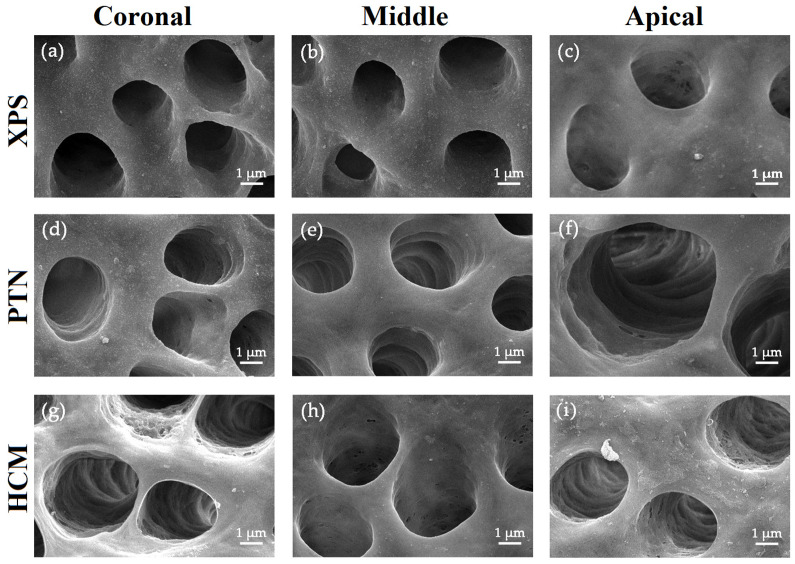
Representative SEM images showing bacteria residue on the buccolingual canal walls after chemomechanical preparation combining PUI in the coronal (**a**,**d**,**g**), middle (**b**,**e**,**h**), and apical thirds (**c**,**f**,**i**) (5000×). No bacteria cells on the surface of the root canal and dentinal tubules. Bars = 1 μm. Abbreviations: HCM: HyFlex CM; PTN: ProTaper Next; PUI: passive ultrasonic irrigation; SEM: scanning electron microscope; XPS: XP-endo Shaper.

For all groups, residue bacteria on the buccolingual canal walls in the upper and middle portions was lower than in the bottom portion. With the application of antimicrobial irrigants, XPS was more effective in eradicating *E. faecalis* in the upper portion of the canals than the other groups (*p* < 0.05) (Figure 4b). When sterile saline or antimicrobial irrigants were used to irrigate the canals, the disinfection of the coronal third using XPS was superior to that of the bottom part (*p* < 0.05). Nonetheless, the use of PUI in the canals did not result in any variation in root canal disinfection among the three groups (Figure 4c and Figure 5).

### 3.3. Assessment of the Effectiveness in Removing the Smear Layer

**Figure 6:**
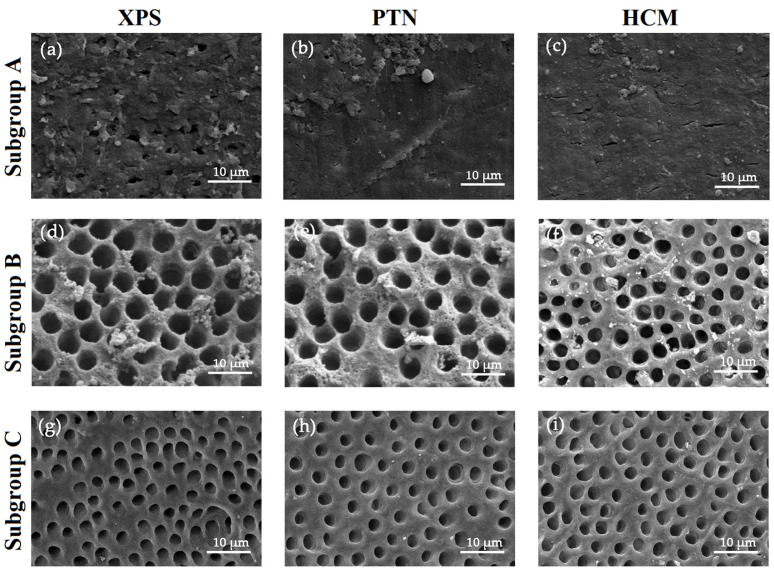
Representative SEM images showing smear layer on the buccolingual canal walls in the coronal third after chemomechanical preparation with different irrigants and rotary systems. (**a**–**c**) Subgroup A: Sterile saline; (**d**–**f**) Subgroup B: 3% NaOCl and 17% EDTA; (**g**–**i**) Subgroup C: 3% NaOCl, 17% EDTA, and PUI. The images are magnified at 1000×. Bars = 10 μm. Abbreviations: HCM: HyFlex CM; PTN: ProTaper Next; PUI: passive ultrasonic irrigation; SEM: scanning electron microscope; XPS: XP-endo Shaper.

When used in combination with PUI, all three rotary instruments demonstrated an improvement in their ability to remove the smear layer on the buccolingual canal walls (*p* < 0.05). In the sterile saline group, the canal walls were heavily covered with a smear layer, and only a few dentinal tubules were open. With the use of 3% NaOCl and 17% EDTA, more dentinal tubules were open, and the canal walls were covered by a small amount of the smear layer. In the PUI group, all dentinal tubules were open, and no smear layer was observed on the surface of the canal walls (Figure 6). In addition, there was no significant difference in the removal of the smear layer among the upper, middle, and bottom parts of the canals when the same rotary or irrigant was applied (*p* > 0.05). The SEM scores of the residual smear layer are presented in Figure 4d–f.

### 3.4. Assessment of the Effectiveness in Cleaning Hard Tissue Debris

The study found that when sterile saline was used for irrigation, XPS showed superior efficacy compared to HCM in cleaning hard tissue debris from the buccolingual canal walls in the upper and middle parts (*p* < 0.05). When canals were prepared with XPS or PTN, the middle third had less debris compared to the apical third (*p* < 0.05). In contrast, when canals were prepared with HCM, the middle third was cleaner than both the coronal and apical thirds (*p* < 0.05), as shown in Figure 4g.

When antimicrobial irrigants were used, XPS and PTN were more effective in reducing debris in the middle portion in contrast to the bottom portion of the canals (*p* < 0.05). However, there were no notable variations in the debris scores of the upper, middle, and bottom portions among three groups, as shown in Figure 4h. 

PUI effectively reduced debris compared to sterile saline irrigation (*p* < 0.05). The combination of XPS and PUI was found to be more effective in removing debris from the middle and bottom parts compared to HCM (*p* < 0.05). XPS achieved better cleaning efficacy by leaving less debris in the middle part of the canal compared to the bottom part, whereas PTN and HCM showed better performance in the upper part of the canal than the bottom part (*p* < 0.05), as shown in Figure 4i.

## 4. Discussion

Effective disinfection through both mechanical and chemical techniques is pivotal for successful root canal treatment [1,2]. It has been well established that oval-shaped root canals are more prone to inadequate cleaning and disinfection along the buccolingual walls as compared to the mesiodistal walls [19,27]. Therefore, it is significant for rotary files to have the ability to adapt to the morphology for oval-shaped root canals [28]. To evaluate the cleaning and disinfecting efficiency of root canals using rotary files, two techniques were used. CFU counts, obtained from samples collected using sterile paper points, were used to measure the number of culturable viable bacterial cells present. Meanwhile, SEM was used to assess the effectiveness of biofilm removal. Since the purposes of the two techniques were not identical, it is not surprising that the results obtained were not completely identical either. Therefore, it is important to consider the different objectives of each technique when interpreting the results, and to recognize that they provide complementary information rather than identical results. The designs and kinematics of rotary files play a vital role in the mechanical disinfection. To evaluate the cleaning and disinfecting efficacy of XPS with adaptive movement, of HCM with traditional continuous rotation, and of PTN with asymmetric movement, this study was specifically conducted on the buccolingual walls of oval-shaped root canals. The findings of this study can provide a valuable perspective on the effectiveness of different instrument designs and kinematics in achieving optimal cleaning and disinfection in oval-shaped root canals.

This study included three rotary systems with the same final apical size, but with different tapers. While XPS can adapt to oval-shaped canals, PTN has a larger taper than both XPS and HCM. Therefore, in theory, there may not be a significant difference in bacterial reduction between XPS and PTN. However, the present study revealed that XPS was more effective in reducing bacterial counts than PTN and HCM when irrigated with sterile saline. This effectiveness may be due to the unique snake-like shape of XPS, which causes the taper to unpredictably expand along the canal length, ranging from 4% to 8%, according to the manufacturer’s statement. A recently published study using Micro-CT analysis reported that XPS had a lower percentage of unprepared areas compared to PTN, which lends support to the present study [19]. While mechanical preparation can remove infected dentin, the eradication of bacteria in dentinal tubules relies on chemical irrigation and disinfecting medication [29]. The study indicated that when combined with an antimicrobial irrigant or PUI, XPS demonstrated greater effectiveness than the other instruments. However, the observed differences were not statistically significant. The findings emphasize the significance of chemical disinfection in root canal debridement and underscore the limitations of solely relying on mechanical instrumentation.

The study demonstrated that when preparing oval-shaped canals, XPS was more effective than other rotary systems in reducing bacterial counts. After instrumentation with XPS, the bacterial counts were reduced by 45.47%, and this percentage increased to 94.98% when combined with PUI. SEM analysis indicated that XPS was more proficient in preparing the upper and middle parts of the buccolingual canal walls, which was in line with a prior study that showed that XPS removed more dentin than Vortex Blue in these areas [11]. However, when compared with other studies on oval-shaped canals prepared with XPS, the bacterial reduction percentages were 80.3% and 86.74% when canals were irrigated with sterile saline, which were higher than the percentages found in the present study [30,31]. The reason for the inconsistencies between our study and previous studies could be attributed to the fact that previous studies utilized distobuccal root canals of maxillary molars and mandibular premolars with straight and round-shaped root canals. These canals, with their regular and narrow shape, allowed XPS to contact a relatively greater surface area of the canal walls during mechanical preparation, resulting in more efficient preparation. In contrast, the long oval-shaped canals in this study had buccolingual extremities that were challenging to disinfect.

It has been well established that hard tissue debris generated during mechanical instrumentation can be packed into the irregular configurations of oval-shaped canals, leading to root canal reinfection from debris containing bacteria [1]. In this study, when irrigated with sterile saline, XPS demonstrated superior ability in removing debris from the buccolingual wall of oval-shaped canals in the upper and middle parts. PTN, which touches the canal wall with two points, enhances the prepared area of canals and provides a larger space for debris extrusion [32]. In the present study, PTN had a similar ability to XPS in removing debris, indicating that the variable tapers of PTN and XPS provide space for debris and that debris suspended in the solution can be effectively removed [23]. The employment of antimicrobial irrigants led to an improved elimination of debris, which aligns with prior research [33,34]. However, in this study, there were no significant differences identified through statistical analysis. PUI removed significantly more debris than conventional needle irrigation with sterile saline, which has also been verified in previous studies [24,35]. This study yielded comparable results and provided evidence that combining XPS with PUI resulted in greater effectiveness than HCM in the middle and bottom parts of the canals. Nonetheless, XPS demonstrated lower cleaning efficacy by leaving a higher amount of debris in the critical apical area of oval-shaped root canals compared to the middle area. Therefore, incorporating advanced irrigation methods such as the GentleWave system following mechanical instrumentation may be considered for enhancing the elimination of apical debris [36]. 

In this study, the ability of XPS, PTN, and HCM to remove the smear layer on the buccolingual canal walls of oval-shaped canals was enhanced when combined with antimicrobial irrigants and PUI, which agrees with the results reported in earlier studies [37,38,39]. Interestingly, although the same rotary system or irrigant was used, there was no significant difference observed in the removal of the smear layer across the entire length of the canals. This finding differs from a published study which reported that the middle and bottom parts of the canal had a greater amount of remaining smear layer compared to the upper part [39]. The cutting capacities and potential to produce a smear layer of rotary files are determined by their designs, alloys, and kinematics, while the removal of a smear layer may depend on chemical irrigation and activation [40]. In light of this, Martins et al. suggested in a comprehensive review that a multimethod research approach, when combined with reliable confounding factor control and appropriate study designs, can be a useful tool and strategy to improve the reliability of study outcomes by enhancing data collection, analysis, and interpretation [41].

This study provides valuable insights into the effectiveness of different cleaning and disinfection techniques for oval-shaped root canals infected with *E. faecalis*, a common bacterial species associated with endodontic infections. The study’s findings can aid clinicians in selecting and optimizing their disinfection and irrigation protocols for better outcomes in oval-shaped root canals. The study highlights that XPS is particularly effective in disinfecting oval-shaped root canals, especially along the buccolingual walls of the upper and middle sections of the canals. This information can guide clinicians in selecting the appropriate instrument for better disinfection in such cases. Additionally, PUI enhances irrigation efficiency by effectively removing bacterial biofilm, hard tissue debris, and smear layer. This information can help clinicians improve their irrigation protocols to achieve better cleaning and disinfection efficacy. The combined use of XPS and PUI further enhances cleaning and disinfection efficacy in oval-shaped root canals. However, the study notes that removing hard tissue debris in the critical apical area remains a challenge. This finding can prompt clinicians to consider alternative techniques or modifications to their existing techniques to address this issue. Nonetheless, it is important to acknowledge that the study’s findings are limited to in vitro settings and may not directly translate to clinical practice. Further clinical studies are necessary to confirm the efficacy of these techniques and strategies in in vivo settings.

Despite stringent inclusion criteria and the meticulous anatomic matching of groups, the small sample size in this study is a limitation that should be acknowledged. It is worth noting that XPS is a relatively new addition to the market, and there is a paucity of studies evaluating its cleaning and disinfecting effects. Hence, the results of this study require further confirmation. In this study, we utilized SEM and paper point sampling techniques to evaluate the efficacy of XPS, PTN, and HCM in combination with PUI for cleaning and disinfecting oval-shaped root canals. While SEM is a valuable tool for assessing bacterial presence and distribution within the root canal, it does have limitations. The process of drying and coating the samples for SEM can potentially alter surface morphology, which may obscure the presence of bacteria. Additionally, SEM’s limited field of view may lead to sampling bias and inaccurate conclusions about bacterial distribution and prevalence. Similarly, paper points, although a widely used technique for bacterial sampling in root canals, have limitations. They have a small surface area and can only sample a limited volume of the root canal, which may not accurately reflect the overall bacterial load in the canal. Furthermore, paper points only sample the surface of the root canal walls and may not reach the dentinal tubules, where bacteria can be present, leading to an incomplete representation of the bacterial population in the canal. Therefore, further study is needed to assess the bacterial biofilm present within the dentinal tubules and confirm the findings of this study using confocal laser scanning microscopy (CLSM) analysis. Another limitation of this study is that the samples were not paired to ensure an even distribution of three-dimensional variables between the groups. Moreover, this study did not investigate the shaping ability of XPS. One of the advantages of XPS is its ability to minimally, invasively, and three-dimensionally clean more surface area of the canal walls [11]. To better validate the minimally invasive but optimal performance of XPS in chemomechanical preparation, future studies should concurrently investigate its shaping ability. A multimethod research approach, when combined with reliable confounding factor control and appropriate study designs, can be a useful tool and strategy to improve the reliability of study outcomes by enhancing data collection, analysis, and interpretation [41]. In this way, we could gain a comprehensive understanding of the instrument’s effectiveness in cleaning, shaping, and the disinfection of oval-shaped root canals. 

## 5. Conclusions

With the limitations of this study, XPS was more effective than PTN and HCM in disinfecting oval-shaped root canals infected with *E. faecalis*, particularly along the buccolingual walls of the upper and middle parts of the canals. PUI enhanced irrigation efficiency, thereby effectively removing bacterial biofilm, hard tissue debris, and the smear layer. While the combined use of XPS and PUI improved the cleaning and disinfecting efficacy in oval-shaped root canals, the removal of hard tissue debris in the critical apical area remained a concern.

## Figures and Tables

**Table 1:** Bacterial counts before and after chemomechanical preparation.

Groups	Sterile Saline		3% NaOCl + 17% EDTA		3% NaOCl + 17% EDTA + PUI	
	S1	S2	S1	S2	S1	S2
XPS	6.29 ± 0.25 ^a^	3.43 ± 0.09 ^b^	6.00 ± 0.45 ^a^	1.73 ± 0.26 ^d^	6.50 ± 0.32 ^a^	0.34 ± 0.55 ^e^
PTN	6.33 ± 0.22 ^a^	4.02 ± 0.16 ^c^	6.12 ± 0.56 ^a^	2.09 ± 0.40 ^d^	6.32 ± 0.28 ^a^	0.75 ± 0.66 ^e^
HCM	6.17 ± 0.25 ^a^	3.99 ± 0.15 ^c^	6.46 ± 0.64 ^a^	2.22 ± 0.27 ^d^	6.60 ± 0.35 ^a^	0.62 ± 0.67 ^e^

Distinct superscript lowercase letters indicate a significant difference between the groups in the line and column being compared (*p* < 0.05, one-way ANOVA). Abbreviations: HCM: HyFlex CM; PTN: ProTaper Next; PUI: Passavie Ultrasonic Irrigation; S1: baseline samples; S2: samples after chemomechanical preparation; XPS: XP-endo Shaper.

**Table 2:** Bacterial counts reduction during chemomechanical preparation.

Groups	Sterile Saline		3% NaOCl + 17% EDTA		3% NaOCl + 17% EDTA + PUI	
	S1–S2	Reduction (%)	S1–S2	Reduction (%)	S1–S2	Reduction (%)
XPS	2.87 ± 0.25	45.47 ^a^	4.27 ± 0.36	71.17 ^c^	6.16 ± 0.44	94.98 ^d^
PTN	2.31 ± 0.28	36.39 ^b^	4.04 ± 0.50	65.97 ^c^	5.57 ± 0.63	88.18 ^d^
HCM	2.18 ± 0.28	35.30 ^b^	4.24 ± 0.63	65.33 ^c^	5.97 ± 0.53	90.86 ^d^

Distinct superscript lowercase letters indicate a significant difference between the groups in the line and column being compared (*p* < 0.05, two-way ANOVA). Abbreviations: HCM: HyFlex CM; PTN: ProTaper Next; S1: baseline samples; S2: samples after chemomechanical preparation; XPS: XP-endo Shaper.

## Data Availability

Not applicable.
